# Does rapamycin slow down time?

**DOI:** 10.18632/oncotarget.25788

**Published:** 2018-07-13

**Authors:** Mikhail V. Blagosklonny

**Affiliations:** Mikhail V. Blagosklonny: Roswell Park Cancer Institute, Buffalo, NY, USA

**Keywords:** rapalogs, mTOR, aging, growth, lifespan

**Figure 1:**
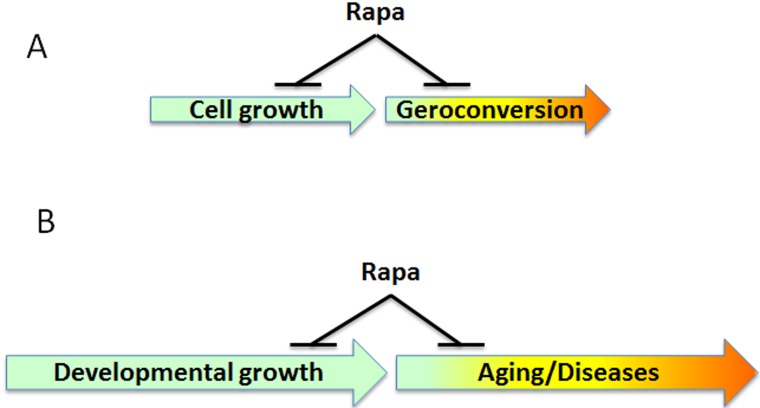
Rapamycin slows aging **A.** Cell culture. In proliferating cells, rapamycin (RAPA) slows growth. When the cell is arrested, then rapamycin slows down geroconversion to senescence. Geroconversion is a continuation of growth in non-dividing (arrested) cells. **B.** The organism. When development is completed, then mTOR drives aging and age-related diseases. Thus, aging and its diseases are quasi-programmed (a continuation of developmental growth). RAPA slows aging and delays diseases.

**Figure 2:**
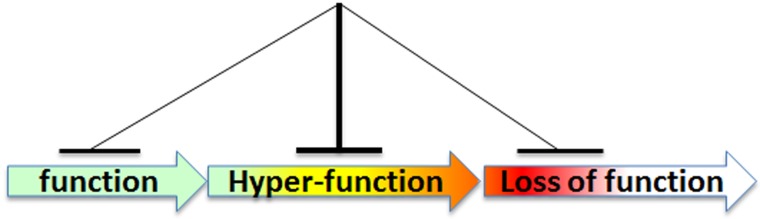
Rapamycin decreases hyper-functions, a key feature of aging, thus preventing functional decline

According to Einstein’s theory, time is relative and can be made to move slower by increasing the speed of the observer. In the famous twin paradox, this slowing of time enables one identical twin to live longer than the other. Animals treated with rapamycin, an inhibitor of mTOR, also live longer [1-5]. Of course, that does not mean that rapamycin slows time in the Einsteinian sense. Instead, it figuratively slows biological time by slowing seemingly opposite processes. On the one hand, rapamycin (and other mTOR inhibitors) retards cell proliferation, while on the other hand, it retards loss of proliferative potential [6, 7]. In other words, rapamycin decelerates proliferation while preserving the potential to proliferate. In that way, rapamycin suppresses both cell growth and geroconversion (conversion to senescence). It has been calculated that rapamycin slows geroconversion by approximately 3-fold [6]. By doing so, rapamycin slows development and aging, reproduction and menopause, and hyperfunction and functional decline [8]. This is because in each case one process is a continuation of the other (Figure 1, 2). For example, aging is a continuation of developmental growth (Figure 1), and functional decline (loss of function, Figure 2) results from earlier hyperfunction [8].

The slowing of biological time entails “suppression plus preservation.” For example, by suppressing beta-cell function, rapamycin preserves beta-cell function in the long run [9-11]; or by suppressing reproduction, it preserves the oocytes, thereby delaying menopause [12-14]. In theory, a woman who wants to have children later in life could postpone reproduction using rapamycin. This can be seen as “freezing the ovaries” until later in life. Rapamycin suppresses cellular hyperfunction and thus delays all diseases of aging, from cancer to Alzheimer’s [8]. Pathological processes such as age-related diseases are continuations (or exacerbations) of physiological processes. Geroconversion is a continuation of growth (Figure 1), hyperfunction is a continuation of tissue-specific cellular function (Figure 2), age-related hypertension and presbyopia are continuations of developmental trends (see Figure 3 in ref. 15). Therefore, aging is both hyperfunctional and quasi-programmed [8, 16, 17]. (A quasi-program is a purposeless continuation of a developmental program.) Hyperfunction eventually leads to organ damage and functional decline [8, 15]. By suppressing hyperfunction, rapamycin delays organ damage (e.g., infarction) and loss of organ function [8, 15]. In addition to their therapeutic effects, the side effects of rapalogs are also consequences of slowing down time. For example, by slowing cell proliferation, high doses of rapamycin induce reversible anemia, mucositis and skin rash.

So why does rapamycin do all that? Rapamycin is produced by the bacterium *Streptomyces hygroscopicus*, which lives in the soil of Easter island [18]. This wonderful microbe had no intention of slowing time on its mysterious island; instead, it sought to slow down the growth of fungi, its natural enemy. But since rapamycin slows growth, it should also slow aging if aging is a continuation of growth. In fact, it does just that; rapamycin prolongs the lifespan of yeast [19].

Rapalogs (rapamycin, everolimus, temsirolimus and deforolimus) are allosteric inhibitors of mTOR complex 1, a central regulator of RNA translation and cellular growth and metabolism [20-23]. mTOR enhances translation of TOP and TOP-like mRNAs [22, 23]. Rapamycin and, especially, the pan-mTOR inhibitor Torin1 slow this translation [22, 23]. This raises the question, is slowing translation equivalent to slowing time and, if so, can biological time be measured based on the speed of translation?

The answer to that question is, not entirely. Although mTOR inhibitors may in principle “slow time” by slowing rapamycin-sensitive translation from mRNA to protein, this does not completely explain the gerosuppressive effects of rapamycin or pan-mTOR inhibitors. In fact, inhibitors of S6 kinase slightly exhibit gerosuppressive activities [24], even though S6 kinase is not crucial for RNA translation [23]. So, mTOR inhibitors may affect the speed of aging by suppressing geroconversion (Figure 1) and cellular function and hyperfunction (Figure 2) independently of TOP mRNA translation. In addition, rapamycin also slows age-related methylation, or the epigenetic clock [25-29].

Another intriguing possibility is that rapamycin slows time by slowing the circadian clock. mTOR inhibition slows the circadian clock and dampens clock oscillations, whereas mTOR activation accelerates the clock and enhances clock oscillations at the level of cells, tissues and mice [30]. Conversely, circadian clock mediators affect the mTOR pathway and aging [31]. Because mTOR activity is itself part of the circadian clock, its sensitivity to rapamycin can vary widely depending on the time of the day and the phase of the clock [32]. This should be taken into account when comparing the numerous studies in mouse models. It should also be taken into account when designing rapamycin-based therapies for aging.

Rapamycin has been combined with several life-extending drugs in the “Koschei” formula [5]. This rapamycin-based drug combination has been successfully used as an anti-aging therapy at the Alan Green clinic https://rapamycintherapy.com

https://roguehealthandfitness.com/rapamycin-anti-aging-medicine-an-interview-with-alan-s-green-m-d/?print=pdf

The older we become, the faster time flies. It is initially measured in days, then in weeks, the four seasons, and finally “Winter-Summer” cycles. Of course, this is an illusion, but an annoying one. Would treatment with rapamycin enable us to notice Spring again?
